# Neuropathological and behavioral features of an APP/PS1/MAPT (6xTg) transgenic model of Alzheimer’s disease

**DOI:** 10.1186/s13041-022-00933-8

**Published:** 2022-06-08

**Authors:** Sung Hyun Tag, Baeksun Kim, Jinhee Bae, Keun-A Chang, Heh-In Im

**Affiliations:** 1 Convergence Research Center for Brain Science, Korea Institute of Science & Technology (KIST), Seoul, 02792 Republic of Korea; 2 Division of Bio-Med (Neuroscience), KIST School, Korea University of Science & Technology (UST), Seoul, 02792 Republic of Korea; 3 Department of Pharmacology, College of Medicine, Gachon University, Incheon, 21999 Republic of Korea; 4 Neuroscience Research Institute, Gachon University, Incheon, 21565 Republic of Korea; 5 Department of Health Sciences and Technology, GAIHST, Gachon University, Incheon, 21999 Republic of Korea

**Keywords:** 6xTg, Alzheimer's disease, Cognitive impairment, Social behavior, Anxiety, Depression

## Abstract

Alzheimer's disease is associated with various brain dysfunctions, including memory impairment, neuronal loss, astrocyte activation, amyloid-β plaques, and neurofibrillary tangles. Transgenic animal models of Alzheimer's disease have proven to be invaluable for the basic research of Alzheimer's disease. However, Alzheimer's disease mouse models developed so far do not fully recapitulate the pathological and behavioral features reminiscent of Alzheimer's disease in humans. Here, we investigated the neurobehavioral sequelae in the novel 6xTg mouse model of Alzheimer's disease, which was developed by incorporating human tau containing P301L mutation in the widely used 5xFAD mouse model of Alzheimer's disease. At 11-months-old, 6xTg mice displayed the core pathological processes found in Alzheimer's disease, including accumulation of amyloid-β plaque, extensive neuronal loss, elevated level of astrocyte activation, and abnormal tau phosphorylation in the brain. At 9 to 11-months-old, 6xTg mice exhibited both cognitive and non-cognitive behavioral impairments relevant to Alzheimer’s disease, including memory loss, hyperlocomotion, anxiety-like behavior, depression-like behavior, and reduced sensorimotor gating. Our data suggest that the aged 6xTg mouse model of Alzheimer's disease presents pathological and cognitive-behavioral features reminiscent of Alzheimer's disease in humans. Thus, the 6xTg mouse model of Alzheimer's disease may be a valuable model for studying Alzheimer’s disease-relevant non-cognitive behaviors.

**Supplementary Information:**

The online version contains supplementary material available at 10.1186/s13041-022-00933-8.

## Introduction

Alzheimer's disease (AD) is a progressive neurodegenerative disorder and the most common cause of dementia in the elderly. AD is characterized by two pathological hallmarks: Amyloid-β plaques and neurofibrillary tangles (NFTs). NFTs are abnormal intracellular aggregates composed of hyperphosphorylated forms of the microtubule-associated protein tau [1]. Tau aggregation is not only directly toxic to neurons but to also a mediator amyloid-β toxicity. Tau aggregation and the presence of NFTs correlate closely with symptom severity and neuron loss [2]. On the other hand, AD-related presenilin mutations can alter intracellular calcium signaling, which leads to amyloid-β aggregation to form amyloid-β plaques and causes neuronal cell death and neuroinflammation in the brain [3, 4]. In the process, reactive astrocytes and activated microglia acquire toxic function and lose neurotrophic function [5].

In the clinic, AD is marked by cognitive impairment including memory deficit [6]. Interestingly, more than 80% of AD patients have been found to exhibit at least one neuropsychiatric symptom since the onset of cognitive impairment, including agitation/aggression, irritability, depression, and anxiety being the most common [7, 8]. In addition, previous studies reported that sensorimotor gating deficit is observed in AD patients [9, 10].

Transgenic mice have proven to be invaluable for modeling and studying various aspects of AD neuropathology [6]. APP/PS1, 3xTg-AD, and 5xFAD mouse models of AD have successfully recapitulated the amyloid-β plaque deposition, generally by attributing the mice with amyloid-β precursor protein (APP) overexpression [11]. Importantly, inclusion of a mutant presenilin-1 (PS1) allele in the 5xFAD mice accelerated the amyloid-β deposition rate as well as exacerbated the pathological severity of amyloid-β plaques [12], offering greater accessibility to the fundamental research on amyloid pathology.

However, the 5xFAD and APP/PS1 do not show the NFT pathology, whereas the 3xTg mice carrying a mutation in microtubule-associated protein tau (MAPT) develop NFT pathology at 6 months of age [13, 14]. Given that NFTs are one of the most vital drivers of cognitive decline in AD, an animal model that can rapidly manifest both the Aβ plaques and NFTs is primarily demanded for the study of AD.

Although the 5xFAD mice do not develop tau pathology, they do show amyloid pathology at 3-months-old [15], rapid onset of cognitive deficit at 4-months-old [12], and mood as well as sensorimotor dysfunctions at 6- to 7-months-old [15, 16]. Such rapid onset of both the neuropathological and behavioral features of AD rendered the 5xFAD mice ideal for studying the mechanisms of AD.

JNPL3 Tg mice overexpress MAPT with P301L mutation under the mouse prion promoter, modeling human frontotemporal dementia with Parkinsonism linked to chromosome 17 (FTDP-17) [14]. The P301L mutation in tau leads to a robust and aggressive phenotype, exhibiting early onset of tauopathy with a high tendency to produce NFTs [17, 18]. Brain insoluble tau accumulation can be observed as early as 5- to 6-months-old of JNPL3 Tg mice [14, 19], while memory deficiency develops at a later age (> 12 months) [20, 21].

Recently, a novel transgenic mouse model of AD with six AD-linked mutations (6xTg) was generated by crossbreeding the 5xFAD mouse model of AD with the JNPL3 mice [19]. NFT pathology was significantly increased in 4-months-old 6xTg mice. Also, the 6xTg mouse model of AD presents cognition deficits at 2 months of age, which is earlier than observed in the 5xFAD [19].

However, the AD-relevant cognitive and non-cognitive behaviors, as well as neuropathological hallmarks, have not been characterized at an older age in the 6xTg mouse model of AD. AD is a neurodegenerative disease whose symptoms manifest mostly at a later age. To grant face validity to any animal model of AD, it is essential to demonstrate that the animal model of AD exhibits cognitive deficit at a later stage of life.

Here, we describe the cardinal phenotypes of the 6xTg mouse model of AD at 9- to 11-months-old. This study aimed to (1) replicate our previous study that the 6xTg mice displays AD-related phenotypes such as pathological changes and cognitive deficits as well as non-cognitive behavioral disturbances, and (2) further validate the 6xTg mouse model of AD by examining the AD-related pathological changes and behavioral characteristics at an older age.

## Result

### AD-related pathological features in the hippocampal formation of the 6xTg mice

The series of analyses were carried out according to the schedule depicted in Additional file 1: Figure S1. In the previous study, AD has been associated with neuropathological correlates in the hippocampal formation: amyloid-β plaque and NFTs. The hippocampal formation includes the hippocampus and entorhinal cortex [22]. First, to investigate the AD-related pathological changes in the hippocampal formation of the 6xTg mice, we examined the degree of amyloid-β plaque formation, neuronal loss, astrocyte activation, and abnormal tau phosphorylation.

**Fig. 1:**
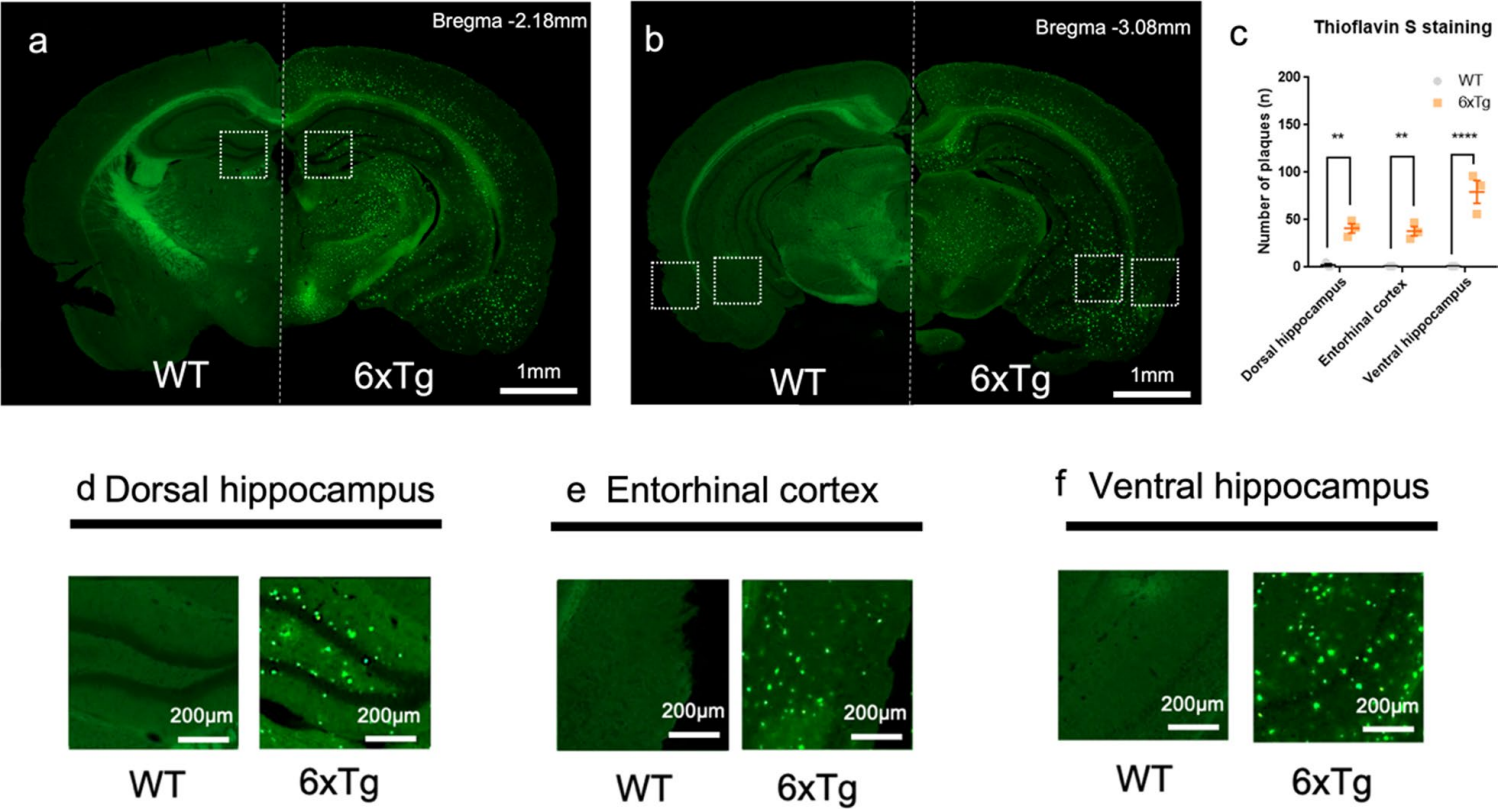
Increase in amyloid-β plaque in the hippocampal formation of the 11-months-old 6xTg mouse model of AD. **a–b** Representative images of Thioflavin S stain in wild-type (WT, left) and 6xTg (right). Representative coronal sections of **c** In the 6xTg mice, the number of plaques was significantly increased compared with WT mice in the dorsal hippocampus, entorhinal cortex, and ventral hippocampus. **d–f** The dorsal hippocampus, entorhinal cortex, and ventral hippocampus from 11-month-old WT and 6xTg mice were stained with Thioflavin S. All data are given as means ± SEM (n = 3 for WT, 3 for 6xTg; One-way ANOVA, **p < 0.01, ****p < 0.0001). Scale bar, 1 mm for **a-b**, and 200 µm for **d-f**

At 11-months-old, the amyloid-β plaque was observed in the brain of the 6xTg mice (Fig. 1a, b). In the 6xTg mice, the number of amyloid-β plaque formation was significantly increased compared with WT mice (One-way ANOVA; **p < 0.01, ****p < 0.0001) (Fig. 1c) in the dorsal hippocampus (Fig. 1d), entorhinal cortex (Fig. 1e), and ventral hippocampus (Fig. 1f).

**Fig. 2:**
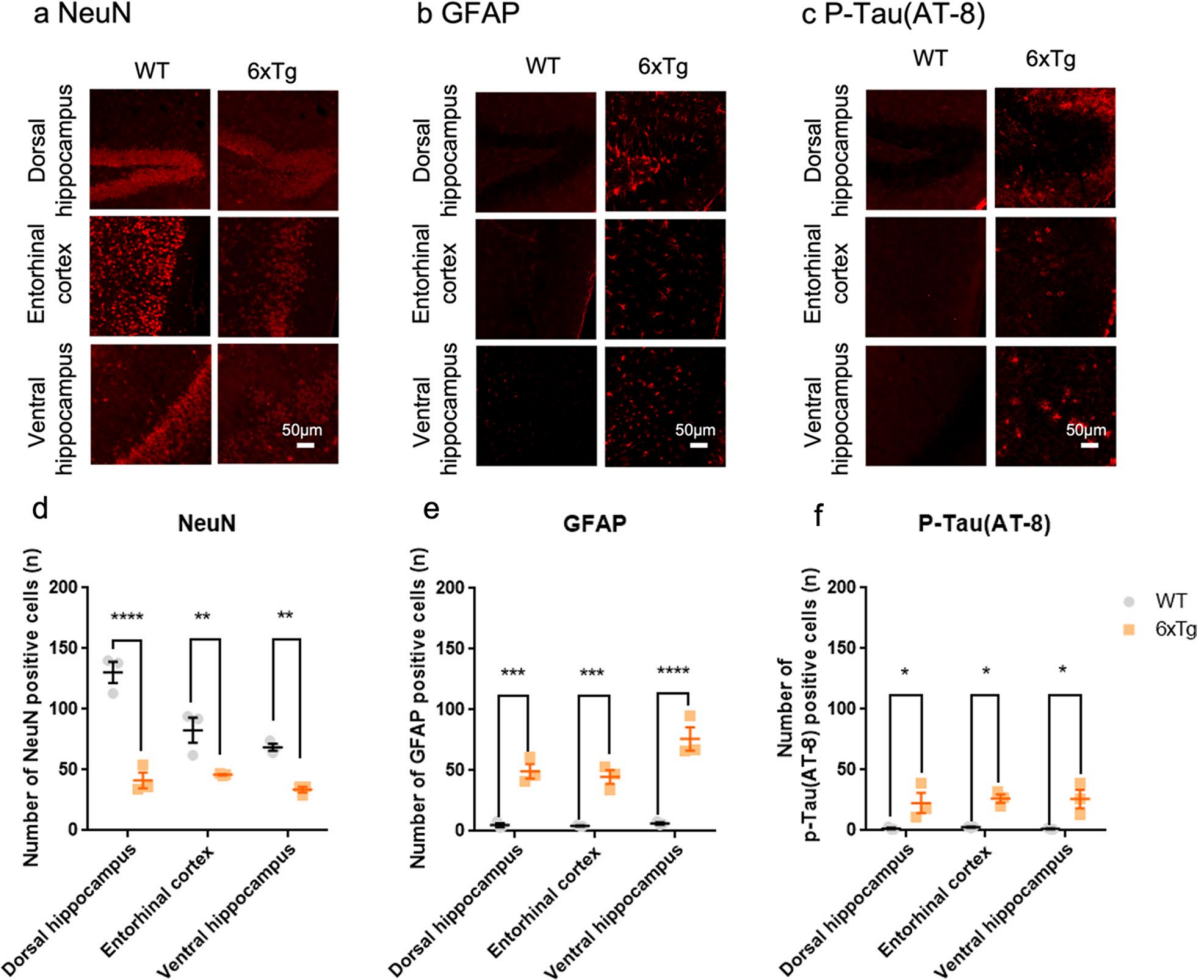
Neuronal loss, astrocyte activation, and formation of neurofibrillary tangles in the hippocampal formation of the 11-months-old 6xTg mouse model of AD. **a–c** Representative coronal sections of the dorsal hippocampus, entorhinal cortex, and ventral hippocampus from 11-months-old WT and 6xTg mice stained with antibodies for NeuN, GFAP, and phosphorylated tau (P-Tau). **d–f** In the 6xTg mice, the number of GFAP positive cells and p-Tau(AT-8) positive cells were significantly increased compared with WT mice, but the number of NeuN positive cells was significantly decreased in comparison with WT mice in the dorsal hippocampus, entorhinal cortex, and ventral hippocampus. All data are given as means ± SEM (n = 3 for WT, 3 for 6xTg; One-way ANOVA, *p < 0.05,**p < 0.01, ***p < 0,001, ****p < 0.0001). Scale bar, 50 µm

Next, we evaluated neuronal and astrocytic changes in the brain of the 6xTg mice. Immunohistochemical analyses revealed extensive neuronal loss, reactive gliosis, and abnormal tau phosphorylation in the dorsal hippocampus, entorhinal cortex, and ventral hippocampus (Fig. 2a–c) of the 6xTg mice. In the 6xTg mice, the number of GFAP positive cells and p-Tau (AT-8) positive cells were significantly increased compared with WT mice, but the number of NeuN positive cells was significantly decreased with WT mice in the dorsal hippocampus, entorhinal cortex, and ventral hippocampus. All data are given as means ± SEM (One-way ANOVA, *p < 0.05,**p < 0.01, ***p < 0,001, ****p < 0.0001) (Fig. 2b–f).

In addition, we also analyzed the neuropathological hallmarks of AD in the 5xFAD mice. At 11-months-old, the 5xFAD exhibited an increase in the amyloid-β plaques, neuronal loss, and reactive gliosis (Additional file 1: Fig. S2a-c, S2e-g). However, unlike in the brain of 6xTg mice, we did not observe a significant increase in the abnormal tau phosphorylation of the brain of the 5xFAD mice (Additional file 1: Fig. S2d, h). These results indicate that the 6xTg mice exhibit the core pathological hallmarks of AD while the 5xFAD mice do not.

### Cognitive impairment in the 6xTg mice

**Fig. 3:**
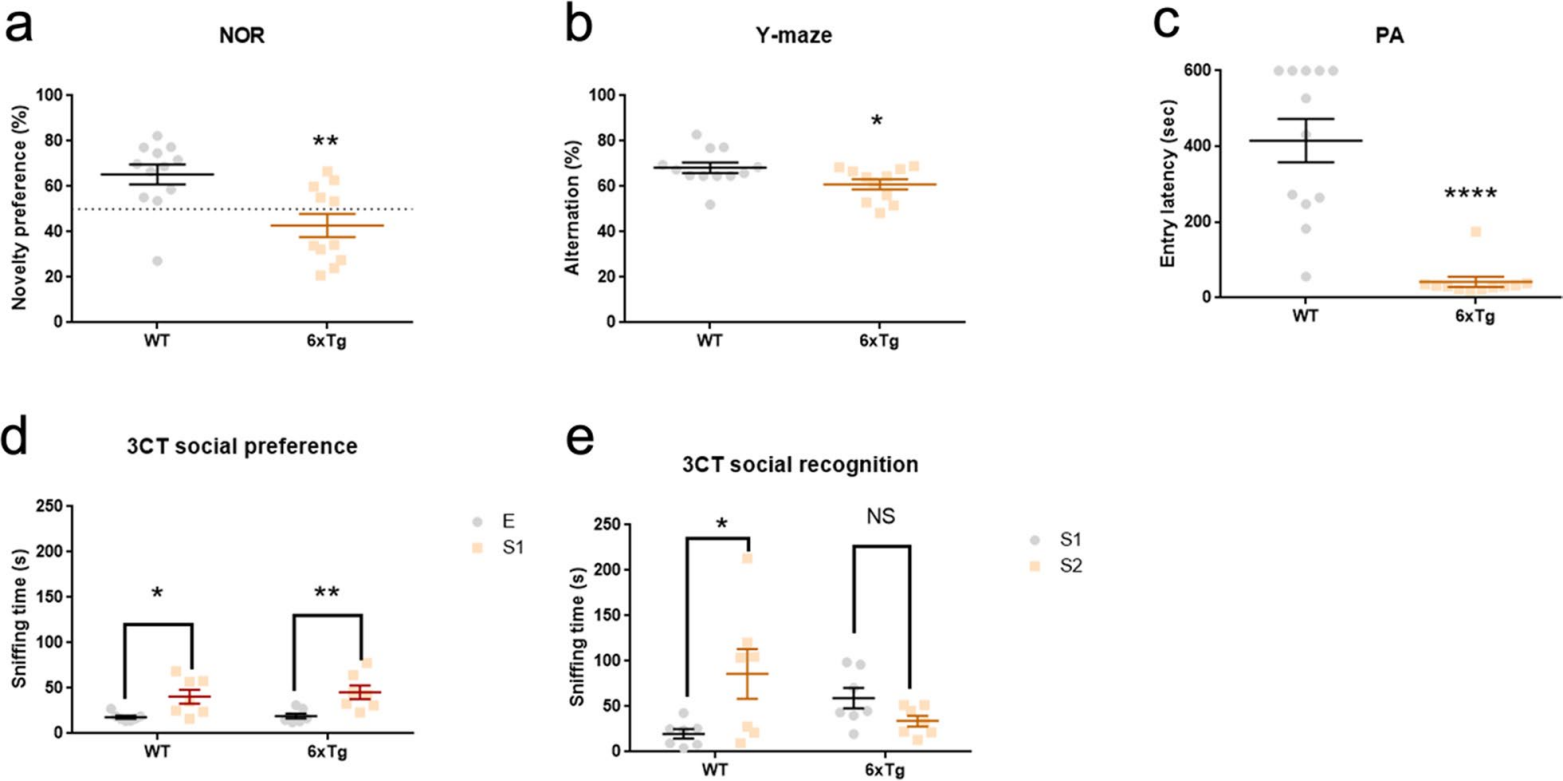
Cognitive deficit in 9- to 11-months-old 6xTg mouse model of AD. **a** 6xTg exhibited reduction in novel object recognition (n = 12 for WT, 11 for 6xTg; Student’s t-test, **p < 0.01). **b** Spontaneous alternation was significantly decreased in the 6xTg compared with that of WT mice (n = 12 for WT, 11 for 6xTg; Student’s t-test, *p < 0.05). **c** The latency to enter the shock-associated chamber was significantly decreased in the 6xTg compared with that of WT mice (n = 12 for WT, 10 for 6xTg; Student’s t-test, ****p < 0.0001). **d** Both the WT and 6xTg mice showed significantly longer sniffing time for the stranger 1 than for the empty cage (n = 7 for WT, 7 for 6xTg; Two-way ANOVA, *p < 0.05, **p < 0.01). **e** WT mice preferred stranger 2, whereas the 6xTg mice did not show preference towards the social novelty (n = 7 for WT, 7 for 6xTg; Two-way ANOVA, *p < 0.05). In the 3CT, data points from 5 WT mice and 4 6xTg mice were omitted as the animals escaped the behavioral apparatus. All data are given as means ± SEM. E, empty; S1, stranger 1; S2, stranger 2

To investigate whether the 6xTg mice recapitulate the cognitive signs observed in AD, we conducted novel object recognition test (NOR), Y-maze test, passive avoidance test (PA), and three-chamber social test (3CT). In NOR, the 6xTg mice failed to recognize the new object as compared to the wild-type mice (WT) (Student's t-test; **p < 0.01) (Fig. 3a). The 6xTg mice also showed a deficit in spontaneous alternation in the Y-maze test (Student's t-test; *p < 0.05) (Fig. 3b). In addition, the 6xTg mice displayed significantly decreased latency to enter the shock-associated chamber in the PA as compared to the WT (Student's t-test; ****p < 0.0001) (Fig. 3c).

Lastly, both the 6xTg and WT mice preferred to explore a conspecific compared to a non-social object during the social preference session of the 3CT (Two-way ANOVA; *p < 0.05, **p < 0.01) (Fig. 3d). However, while WT showed a preference for a novel stranger mouse over a familiar mouse, 6xTg mice did not display this preference, indicative of social recognition deficits (Two-way ANOVA; *p < 0.05) (Fig. 3e). These findings demonstrate that the 6xTg mice can model cognitive impairment observed in AD.

### Hyperlocomotion in the 6xTg mice

In the open field test (OF), the 6xTg mice showed significantly increased locomotor activity as compared to the WT (Two-way ANOVA; *p < 0.05) (Additional file 1: Fig. S3a), but no significant difference was observed for the time spent in the center zone between the 6xTg and WT mice (Additional file 1: Fig. S3b).

### The emergence of anxiety-like and depression-like behaviors in the 6xTg mice

**Fig. 4:**
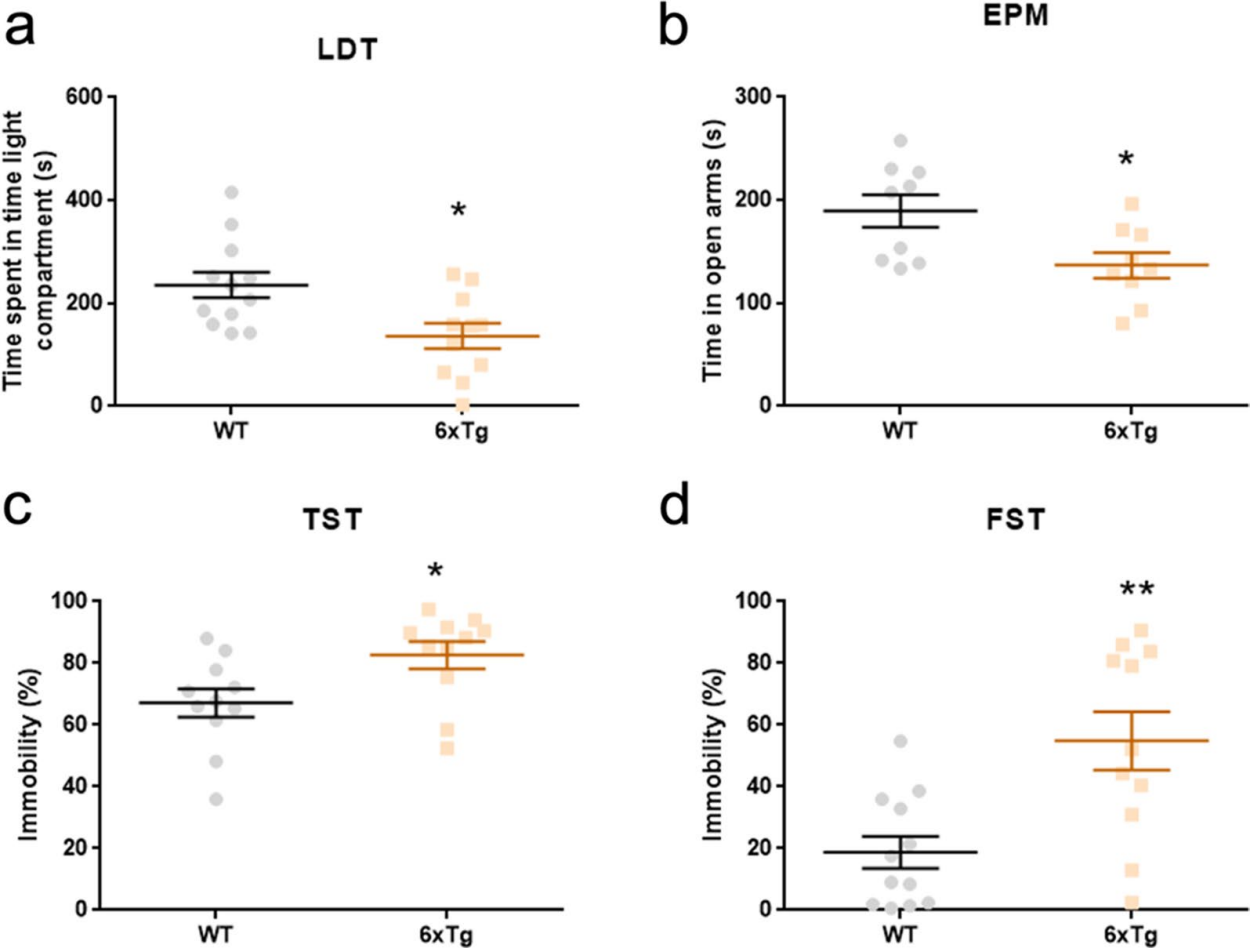
Increase in anxiety-like and depression-like behaviors in the 9-months-old 6xTg mouse model of AD. **a** On the light–dark box test, the 6xTg spent significantly less time in the light compartment than WT (n = 12 for WT, 11 for 6xTg; Student’s t-test, *p < 0.05). **b** On the elevated plus-maze test, the 6xTg spent significantly less time in the open arms than WT (n = 9 for WT, 9 for 6xTg; Student’s t-test, *p < 0.05). Data points from 3 WT mice and 2 6xTg mice were omitted as the animals fell from the behavioral apparatus. **c–d** In forced swimming test and tail suspension test, the 6xTg displayed increased duration of immobility compared to WT (n = 11 for WT, 11 for 6xTg; Student’s t-test, *p < 0.05, **p < 0.01). In the TST, a data point from 1 WT mouse was omitted as the animal climbed up on its tail. All data are given as means ± SEM

Next, we assessed in the 6xTg mice the neuropsychiatric symptoms commonly observed in AD. In the light–dark box test (LDT), the 6xTg mice spent significantly less time in the light compartment (Student's t-test; *p < 0.05) (Fig. 4a), and made significantly less number of entries into the light compartment in the LDT (Additional file 1: Fig. S4e). Interestingly, the 6xTg mice exhibited hyperactivity in the light compartment in the LDT (Additional file 1: Fig. S4f), which is similar to the results in the OF.

On the other hand, the 6xTg mice tended to avoid the open arms and spent more time in the closed arms in the elevated plus-maze test (EPM) (Student's t-test; *p < 0.05) (Fig. 4b). In addition, the number of entries made to each arm in the EPM did not significantly differ between the 6xTg and WT mice (Additional file 1: Fig. S4g, h).

Subsequently, in both the forced swimming test (FST) and tail suspension test (TST), we found that the 6xTg mice displayed a higher level of immobility in comparison to the WT (Student's t-test; *p < 0.05, **p < 0.01) (Fig. 4c, d). These data demonstrate that the 6xTg mice exhibit depression-like behavior.

### Sensorimotor gating dysfunction in the 6xTg mice

**Fig. 5:**
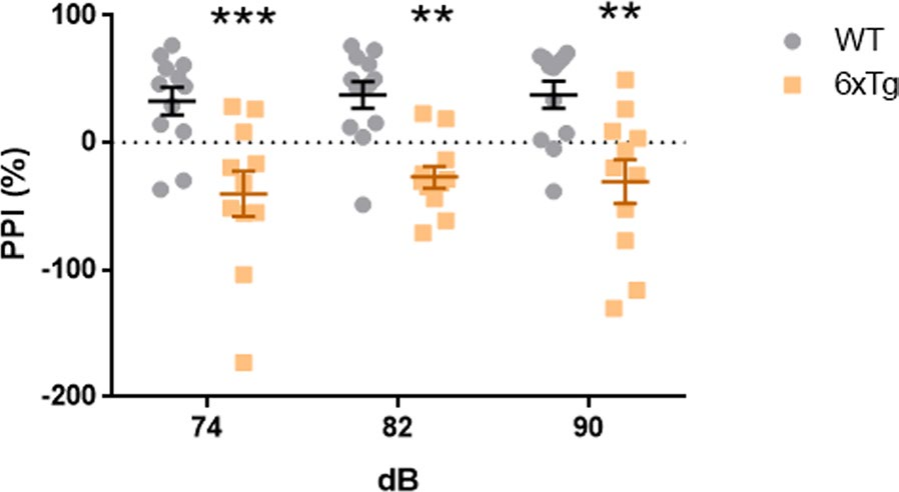
Impaired sensorimotor gating in the 9-months-old 6xTg mouse model of AD. In the prepulse inhibition test, the 6xTg mice showed decrease in prepulse inhibition rate compared to WT (n = 12 for WT, 11 for 6xTg; Two-way ANOVA, **p < 0.01, ***p < 0.001). All data are given as means ± SEM

In the previous studies, sensorimotor gating dysfunction was detected in AD patients [9, 10]. We performed prepulse inhibition test (PPI) to investigate the sensorimotor gating deficit in the 6xTg mice. We found that the 6xTg mice exhibited decreased PPI rate across a range of prepulse intensity (74, 82, and 90 dB above the background noise) as compared to the WT (Two-way ANOVA; **p < 0.01, ***p < 0.001) (Fig. 5). These findings suggest that the 6xTg mice exhibit a sensorimotor gating deficit.

## Discussion

The present study found that the 11-months-old 6xTg mice showed the core pathological processes found in AD, including accumulation of amyloid-β plaque, extensive neuronal loss, elevated astrocyte reactivity, and abnormal tau phosphorylation in the brain. Furthermore, the 6xTg mice exhibited memory loss, hyperlocomotion, anxiety-like behavior, depression-like behavior, and reduced sensorimotor gating at 9 to 11 months of age.

### JNPL3 background in the 6xTg mouse model of AD

JNPL3 homozygous mice exhibit visual dysfunction due to the Pde6b^rd1^ retinal degeneration allele [14]. We did not screen for the 6xTg mice that are homozygous for the Pde6b^rd1^ retinal degeneration allele, which could confound the results of the behavioral assays that depend on visual function. Still, several behavioral parameters indicate that the 6xTg mice were not blind at the time of behavioral experimentation.

First, the 6xTg mice showed heightened anxiety-like behavior in the LDT and EPM. However, mice with retinal degeneration likely show reduced anxiety-like behavior [23] evidently due to the visual cues (light in the LDT and height in the EPM). Second, the entry numbers for the LDT and 3CT were similar between the wild-type and 6xTg mice. As the apparatus contains a small hole placed on the center of a wall, the mice should depend on visual guidance to locate the hole, and therefore the mice with visual impairment would have had a lower number of entries. These observations collectively indicate that the 6xTg mice were not likely blinded at 9–11 months of age.

Lastly, the Y-maze test was suggested to be independent of retinal degeneration [24], and our result indicated that the 6xTg exhibited cognitive dysfunction in the Y-maze test. This finding suggests that the cognitive deficit in the 6xTg mice is likely independent of visual function.

### Pathological changes in the 6xTg mouse model of AD

The 6xTg mice developed an AD-relevant neuropathological phenotype, including amyloid-β plaque formation, neuronal loss, astrocyte activation, and abnormal tau phosphorylation. These pathological changes appeared in the hippocampal formation brain structures most profoundly impacted by the AD pathology. In the previous study, the entorhinal cortex has been shown to play a role in sensorimotor gating [10, 25, 26]. The dorsal hippocampus is responsible for cognitive processing, such as spatial learning [27]. Additionally, neuronal loss of this brain region has consistently been found to be correlated with depression [28]. Lastly, the ventral hippocampus has been shown to play essential roles in anxiety [29] and social memory [30, 31]. These findings suggest that the pathological changes in these brain regions could be responsible for the behavioral deficits.

However, caution must be taken in the interpretation of the potential relationship between behavior and tau pathology. For instance, a mounting number of previous studies demonstrated that mice expressing human mutant Tau exhibited heightened anxiety [32–37]. On the other hand, other studies showed that mice with human mutant Tau displayed normal anxiety-like behavior [38–40] or even reduced anxiety [23, 41–46]. The overall data suggest that Tau mutation can modulate behavior in a complex manner.

In the instance of anxiety-like behavior, we found that introduction of P301L mutant Tau into the 5xFAD mice results in the emergence of increased anxiety-like behavior, which suggests that the interaction between the P301L mutant Tau and the underlying genetic defects could aberrantly regulate the anxiety-related brain regions (e.g. the amygdala, prefrontal cortex, and ventral hippocampus) [47]. For instance, here we demonstrated that the phosphorylated tau was significantly increased in the ventral hippocampus, suggesting that the limbic system was likely compromised by NFTs in the 6xTg mice. However, the potential differences in neuronal activity, stress responses, and the role of Tau within the anxiety-related brain regions remain unexplored. Further studies are warranted to shed light on the brain mechanism underlying why 6xTg mice exhibit heightened anxiety while 5xFAD mice show reduced anxiety.

Accordingly, the relationship between neuropathological hallmarks (especially the tau pathology) and other behavioral phenotypes is essential to support our conclusion that the 6xTg mice are useful for studying the mechanism of AD.

### Cognitive and non-cognitive behavioral changes in the 6xTg mice

Memory loss is the most common problem in AD patients [48]. In the previous study, memory deficits occurred in 6xTg mice at 2 months of age [19]. In our study, we showed that the 6xTg mice developed cognitive behavioral deficits as observed in the NOR, Y-maze, PA, and 3CT at 9–11 months. Previous studies showed that APP/PS1 mice display learning and memory deficits at 7 months of age [49] and 5xFAD mice at 4–5 months of age [12]. In the 3CT, 6-months-old APP/PS1 mice showed decreased social recognition [50], but the 5xFAD mice did not show social recognition deficit [51]. Overall, we demonstrated that the 6xTg mice exhibit cognitive deficits, similar to the results observed in the APP/PS1 and 5xFAD mice model of AD.

Our investigation revealed that the 6xTg mice exhibit significantly increased anxiety paired with increased locomotion. 7-months-old APP/PS1 mice also showed increased anxiety levels [52], whereas 9-months-old 5xFAD mice showed decreased anxiety levels [53]. In addition, the locomotor activity was increased in 6-months-old APP/PS1 mice [54] while it was reduced in 9-months-old 5xFAD mice [53]. The conflicting findings on anxiety-like behavior and locomotor activity among the animal models of AD warrant further study.

Furthermore, the 6xTg mice showed depression-like behavior in the FST and TST. Similar results were observed in 6-months-old APP/PS1 mice [55], whereas 6-months-old 5xFAD mice did not develop depression-like behavior [56]. Lastly, AD patients commonly exhibit sensorimotor gating dysfunction in the PPI test [10]. In accordance, the 6xTg mice showed a significantly lower level of PPI. Similar results were seen in the 5xFAD mice. In a previous study, hearing impairment was observed in 5xFAD mice at 12 months of age. This suggests that the salience of tones previously used in fear conditioning may be reduced in 5xFAD mice, impairing acquisition of the tone-shock fear memory [16]. Further research is therefore needed to characterize the neuropathology within the brain regions mediating both the auditory function and the sensorimotor gating in the 6xTg mice. In sum, 6xTg mice showed both sensorimotor gating deficit and depression-like behavior in line with the clinical and preclinical studies.

### The difference in anxiety-like behavior in the OF, LDT, and EPM

The 6xTg mice exhibited a lack of anxiety-like behavior in the OF but manifested anxiety-like behavior in the LDT and EPM. This might be attributed to the difference in the behavioral tests.

It should be noted that the anxiogenic level in the OF and LDT differs from that in LDT and EPM, as the latter apparatuses provide shelter to where the mice can escape [57, 58]. Usually, mice with high anxiety tend to stay longer in the sheltered zone where they could protect themselves. In the OF, mice have nowhere to escape, hence the anxiogenic levels of the center zone and the periphery may not be significantly different. However, the EPM has a closed arm while the LDT has a dark compartment, both of which create the sheltered zone in which mice can escape from the anxiogenic area. Therefore, anxiety-related behavior may be more reliably measured in the EPM and LDT than in the OF.

### The difference in exploratory behavior in the OF and LDT with NOR and 3CT

The distance covered by the 6xTg in the OF and in the light compartment of LDT were significantly higher in the 6xTg mice, which indicates that the 6xTg exhibits hyperactive phenotype.

Both the OF and the light compartment in the LDT can be considered as an “open arena” where mice can explore rather freely. However, the hyperactive phenotype emerged only in the OF and LDT while it did not in the NOR and 3CT, the other behavioral assays with an open arena. The lack of hyperactivity in the NOR and 3CT implies that the hyperactivity in the 6xTg mice is environment-dependent.

In the NOR and 3CT, mice explore in the presence of salient stimuli (objects and conspecifics) that obligates the mice to examine. In the OF and LDT, such salient stimuli do not exist in the open arena. In such an environment with the absence of salient stimuli, the 6xTg mice might be exhibiting compensatory hyperactivity due to low arousal, which is a phenotype that can be observed in ADHD [59].

It is important to note that the symptoms of ADHD closely resemble that of mild cognitive impairment [60–62], a precursor phenotypic expression of AD. In such a case, it could be inferred that the 6xTg mice model of AD might also exhibit attention deficit that supplements the mild cognitive impairment. Further studies should follow in order to clarify whether the 6xTg mice do exhibit other symptomatic features of ADHD.

### Advantages of the 6xTg mice over other mouse models of AD

First, the onset of cognitive deficit in the 6xTg mice is at 2 months of age [19], suggesting that the 6xTg mice undergo the earliest cognitive decline compared to the other major mouse models of AD (5xFAD, 3xTg-AD, APP/PS1). Second, the 6xTg mice develop both the NFTs and amyloid-β plaques, which are recapitulated only in the 3xTg-AD mice [13]. However, our previous study demonstrated that the onset of neuropathological hallmarks is substantially earlier in the 6xTg mice than in the 3xTg-AD [19]. These points suggest that the 6xTg mice are a valuable asset for studying the potential role of tau accumulation and NFTs in cognitive deficit.

In addition, this study showed that the aged 6xTg mice exhibited mood disturbance and sensorimotor dysfunction as observed in other mouse models of AD [15, 55, 63, 64], yet the time onset of non-cognitive behavioral dysfunction was not investigated. The accelerated onset of cognitive deficit in the 6xTg mice suggests that the time of onset for mood disturbance and sensorimotor dysfunction could also have accelerated. Further studies are required to clarify this notion.

### Limitations

The limitation of this study is the absence of the study on AD-relevant non-cognitive behaviors at a younger age in the 6xTg mouse model of AD. The immediate follow-up study should subdivide the time point and conduct relevant experiments to examine the time course of neuropathological development as well as the possibility of progressive deterioration of learning & memory in the follow-up study.

Additionally, the potential additive and interactive effects of multiple gene mutations in 6xTg mice have not been explored. Future studies should uncover how the aberrant genetic interactions in 6xTg mice give rise to the acceleration of the pathological and behavioral hallmarks, and the potential existence of the progressive worsening of the AD phenotypes. A collective observation of AD and non-AD phenotypes simultaneously in both young and old 6xTg mice should be conducted in conjunction with the experimental schemes to probe the causal relationship between gene and behavior.

Also, the neurobiological mechanisms underlying the AD-relevant non-cognitive behaviors and how they may interact with the cognitive symptoms of AD remain poorly understood. In the future, additional experiments to address these questions could further improve our understanding of AD pathophysiology.

Lastly, future studies should clarify the relationship between visual functioning and the Pde6b^rd1^ blindness allele in the 6xTg mice in order to clarify their potential impact on behavior.

## Conclusion

The 11-months-old 6xTg mice displayed the central pathological processes found in Alzheimer's disease, including accumulation of amyloid-β plaque, extensive neuronal loss, elevated level of astrocyte activation, and neurofibrillary tangles in the brain. Furthermore, the 9–11 months-old 6xTg mice exhibited memory loss, hyperlocomotion, anxiety-like behavior, depression-like behavior, and dysfunctional sensorimotor gating. These findings collectively replicate and further validate the 6xTg mice as a mouse model of Alzheimer’s disease. We conclude that the 6xTg mouse model of Alzheimer's disease is an animal model suitable for studying the impact of amyloid-β and tau pathology observed in Alzheimer’s disease as well as the neurobiological mechanisms underlying the Alzheimer’s disease-relevant impairment in cognitive and non-cognitive behaviors.

## Methods

### Animals

We used 5xFAD (B6SJL) Tg mice (The Jackson Laboratory, ME, USA) overexpressing mutant human APP K670N/M671L + I716V + V717I and mutant human PS1 M146L + L286V [12]. We also used 6xTg (B6SJL) Tg mice (The Gachon University, The Republic of Korea), in which hemizygous 5xFAD transgenic mice were crossbred to JNPL3 hemizygous transgenic mice [19]. Genotyping was performed by ear snipping, DNA isolation, and polymerase chain reaction (PCR) with the primers of PS1, APP, and MAPT. Wild-type littermates were used as control mice. 2–3 mice were group-housed with mixed genotype in a plastic cage (220 × 280 × 130 mm; OrientBio, Gyeonggi-do, South Korea) containing shredded aspen shaving (N323; NEPCO, Warrensburg, NY, USA). Mice had free access to a regular diet (#5053; LabDiet, Richmond, IN, USA) and purified water. Mice were maintained on a 12/12 h dark/light cycle (lights on at 6:00 p.m.) in a climate-controlled vivarium (22 °C). Before the experiment, mice were handled daily for three days. The daily behavioral observation took place between 10 a.m. and 5 p.m. Mice were habituated to the procedure room for 30 min before the beginning of each behavioral test. All experiments were done blind with aspect to the genotype of mice. Only male mice were used throughout experiments. All experimental procedures were approved and conducted following the Institutional Animal Care and Use Committee (IACUC) guidelines of the Korea Institute of Science and Technology.

### Brain preparation

Eleven-month-old mice were anesthetized by intraperitoneal avertin (250 mg/kg) injection, perfused transcardially with 1X PBS followed by 4% of formaldehyde in PBS, and the brain was isolated and post-fixed for overnight. The fixed brain was then immersed in 30% sucrose in PBS and stored at 4 °C for 3 days. Brains were frozen in OCT compound with dry ice and cut into 40 μm serial coronal sections. Sections were stored at − 20 °C in glycerol-PBS (1:1). For Thioflavin S staining and immunohistochemistry experiments, brain sections were collected from the same pre-defined regions within the dorsal hippocampus (AP = − 2.18 mm, ML =  ± 1.0 mm, DV = − 2.0 mm), entorhinal cortex (AP = − 3.08 mm, ML =  ± 3.5 mm, DV = − 3.5 mm), and Ventral hippocampus (AP = − 3.08 mm, ML =  ± 3.5 mm, DV = − 3.5 mm).

### Thioflavin S staining

40 μm-thick brain sections were washed with 1X PBS and dehydrated with graded alcohol solutions (70%, 80% for 1 min each). This was followed by incubation with 1% thioflavin-S (Sigma Aldrich, T1892) solution in 80% ethanol for 15 min in the dark. Sections were rehydrated with graded alcohol solutions (80%, 70% for 1 min each) in the dark. Then, sections were mounted onto glass slides, air-dried, and coverslipped with an aqueous mounting medium. Analysis of plaque counting was performed by using ImageJ software.

### Immunohistochemistry

Brain sections were prepared for free-floating immunohistochemical staining. The sections were blocked with 5% normal donkey serum and then incubated for 48 h at 4 °C with the primary antibody to Mouse Anti-NeuN (1:200, Millipore, mab377), Mouse Anti-GFAP (1:200, Santa Cruz, sc-33673) and Mouse Anti-AT-8 (1:200, Thermo Fisher Scientific MN1020). The sections were then incubated for 2 h at room temperature with a corresponding Donkey Anti-Mouse Alexa fluor 594-conjugated secondary antibody (1:400, Invitrogen, A-21203). The sections were mounted and imaged on a confocal laser scanning microscope. Analysis of cell counting was performed by using ImageJ software.

### Open field test (OF)

Locomotor activity was measured using the open field test. Mice were individually placed in a white square box (40 × 40 × 40 cm, Center: ~ 5 lx; Periphery: ~ 4 lx) by facing the wall and kept for 30 min. The locomotor activity and center duration of the mice were videotaped and analyzed by the Noldus EthoVision XT video tracking system.

### Novel object recognition test (NOR)

Cognitive function was measured using the novel object recognition test. In the 10-min training trial (familiarization phase), mice were allowed to explore two identical objects, to which they had been previously habituated for 10 min, in a white square box (40 × 40 × 40 cm, Center: ~ 5 lx; Periphery: ~ 4 lx). In the 10-min testing trial (recognition phase) performed 24 h after the training trial, mice were allowed to explore two different objects, one familiar object and one new object that was different in color and shape from the familiar object. The sniffing time for the familiar (old) or the new object (novel) during the test phase was videotaped and manually analyzed.

### Y-maze test (Y-maze)

Spontaneous alternation performance was measured using the Y-maze test. Mice were placed in the center of the Y-maze test apparatus (35 cm × 5 cm × 16 cm, ~ 5 lx). Mice were allowed to explore freely throughout the maze for 10 min. The sequence and the total number of arm entry were video-recorded. An arm entry was considered complete when the mouse's hind paws had been entirely placed in the arm. Alternation percentage was calculated as the number of spontaneous alternations (in which mice entered into all three arms consecutively) divided by the maximum possible alternations (the total number of arms entered minus 2) multiplied by 100%.

### Passive avoidance test (PA)

Long-term fear memory was measured using the passive avoidance test. The test apparatus consisted of light (~ 100 lx) and dark compartments separated by a gate. Mice were placed in the light compartment, and the gate was opened after 1 min. When the mice entered the dark compartment, the gate was closed, and an electrical foot shock (0.45 mV, 2-s duration) was applied. Mice were left in the dark compartment for 1 min after the foot shock to enable association of the environment with the aversive stimulus. Mice were then returned to their home cages. The next day, mice were placed in the light compartment again, and the gate was opened after 1 min. The latency to enter the dark compartment was measured, with the maximum latency of 600 s.

### Three-chamber social test (3CT)

Social preference and social recognition were assessed using a modified version of the three-chamber social test [65, 66]. The test arena consisted of three adjacent chambers (60 cm × 40 cm × 20 cm, ~ 10 lx) separated by two clear plastic dividers and connected by an open doorway (4 cm × 3 cm). The test consisted of three 10-min sessions without inter-trial intervals. In the first session, subject mice were habituated to the arena and freely investigate the three chambers. In the social preference session, a never-before-met male mouse (stranger 1) was placed under a metal grid (15 cm height × 7 cm diameter solid bottom with stainless steel bars spaced 1 cm apart) in one of the side chambers and another identical empty metal grid was placed in the other side chamber. In the social recognition session, the empty metal grid was replaced by stranger 1 in a new metal grid. A second never-before-met male mouse (stranger 2) was placed at the previous position of stranger 1 under a new metal grid. The time spent sniffing each metal grid was scored.

### Light–dark box test (LDT)

The tendency to explore a novel, lighted area was assessed using the light–dark box test. The apparatus consisted of an acrylic box (27 cm × 45 cm × 27 cm) divided into light (27 cm × 27 cm × 27 cm, ~ 10 lx) and dark (27 cm × 18 cm × 27 cm) compartments. Mice were placed in the dark compartment, connected to the light compartment by a small hole (7 cm × 7 cm). Mice were allowed to freely explore the apparatus for 10 min. The time spent in the time light compartment was measured using the Noldus EthoVision XT video tracking system.

### Elevated plus-maze test (EPM)

Anxiety-like behavior was assessed using the elevated plus-maze test. The elevated plus-maze apparatus consisted of an elevated maze with four arms (60 cm × 10 cm each); two open arms and two black closed (18 cm high wall) arms, connected by a central square (10 cm × 10 cm, ~ 100 lx). The maze was elevated 50 cm from the ground. Mice were placed in the central square facing the closed arms and allowed to freely explore the maze for 10 min. The whole behavioral session was video-recorded and the time spent in each compartment (open arms, closed arms, or center zone) was manually analyzed. Entry to a compartment was defined as three paws of a mouse laid on the compartment.

### Forced swimming test (FST)

Depression-like behavior was assessed using the forced swimming test. Mice were placed individually in a clear plastic cylinder (25 cm height × 15 cm diameter) that contained 15 cm of fresh water at 26 ± 0.5 °C. The mice were exposed to a swimming session for 6 min. The whole behavioral session was video-recorded and the mobility state was manually analyzed. The rate of immobility (floating in the water without struggling and making minimal movement necessary to float) was measured within the last 4 min of the session.

### Tail suspension test (TST)

Depression-like behavior was assessed using the tail suspension test. Mice were suspended by tethering the extremity of their tail to metallic gallows with adhesive tapes. The head of mice was placed at the height of 30 cm from the ground. The mice were considered immobile only when they were entirely motionless. The immobility rate was measured using the Noldus EthoVision XT video tracking system.

### Prepulse inhibition test (PPI)

Sensorimotor gating was evaluated using the prepulse inhibition test. A mouse restraining cylinder (9 cm × 3 cm diameter) was mounted on an isolation chamber, which measured vibrations produced by movements of the mouse. Background noise was set at 65 dB. Five types of trials were used. Pulse-alone trials (P) consisted of a single white noise burst (120 dB, 40 ms). The prepulse + pulse trials (PP74P, PP82P, and PP90P) consisting of a prepulse of noise (20 ms at 74, 82, or 90 dB, respectively) followed 100 ms after prepulse onset by a startling pulse (120 dB, 40 ms). No-stimulus (NS) trials consisted of background noise only. Sessions were structured as follows: (1) 10-min acclimation at background noise level; (2) five P trials; (3) ten blocks each containing all five trials (P, PP74P, PP82P, PP90P, and NS) in pseudorandom order; and (4) five P trials. Inter-trial intervals were distributed between 12 and 30 s. The average percent reduction in startle intensity between pulse and prepulse + pulse trials at all three prepulse levels was defined as the PPI level. The percent PPI was calculated using the formula [1 − (V_max_ of PPI trials/V_max_ of startling alone trials)] × 100%. Startle magnitude was calculated as the average response to all of the P trials, excluding the first and last blocks of five P trials.

### Statistical analysis

Data represent the mean ± standard error of the mean (SEM). GraphPad Prism 6.0 software was used for statistical analysis. Mice that escaped from the behavioral apparatus or failed to finish the behavioral test due to experimenter error were excluded from analysis. Differences between the two groups were analyzed using the independent sample t-test. Group differences were detected using either one-way analysis of variance (ANOVA) followed by Bonferroni post-hoc test or two-way ANOVA followed by Bonferroni post-hoc test. Differences were considered statistically significant at *p < 0.05. **p < 0.01, ***p < 0.001, and ****p < 0.0001.

## Supplementary Information

**Additional** **file 1.** **Figure S1.** The experimental schedule. **Figure S2.** Alzheimer’s disease-related pathological phenotypes in the hippocampal formation of the 5xFAD mice at 11 months of age. **Figure S3.** Increase in the general locomotor activity in 9-months-old 6xTg mice in the OF. **Figure S4.** Generally unchanged activity during the cognitive and anxiety tests in the 6xTg mice.

## Data Availability

Datasets supporting the findings of this paper are available from the corresponding author upon reasonable request.

## Abbreviations

AD: Alzheimer's disease
NFTs: Neurofibrillary tangles
MAPT: Microtubule-associated protein tau
APP: Amyloid-β precursor protein
PS1: Presenilin-1
NOR: Novel object recognition test
PA: Passive avoidance test
3CT: Three-chamber social test
LDT: Light–dark box test
EPM: Elevated plus-maze test
OF: Open field test
FST: Forced swimming test
TST: Tail suspension test
PPI: Prepulse inhibition test
