# Supplementary material for: Neuropathological and behavioral features of an APP/PS1/MAPT (6xTg) transgenic model of Alzheimer’s disease
Source: Mol Brain. 2022 Jun 8;15:51. doi: 10.1186/s13041-022-00933-8 (PMC9175339; doi:10.1186/s13041-022-00933-8)
Supplement: Supplementary file 1 — Additional file 1. Figure S1. The experimental schedule. Figure S2. Alzheimer’s disease-related pathological phenotypes in the hippocampal formation of the 5xFAD mice at 11 months of age. Figure S3. Increase in the general locomotor activity in 9-months-old 6xTg mice in the OF. Figure S4. Generally unchanged activity during the cognitive and anxiety tests in the 6xTg mice. [file 13041_2022_933_MOESM1_ESM.docx]

**Additional file 1**


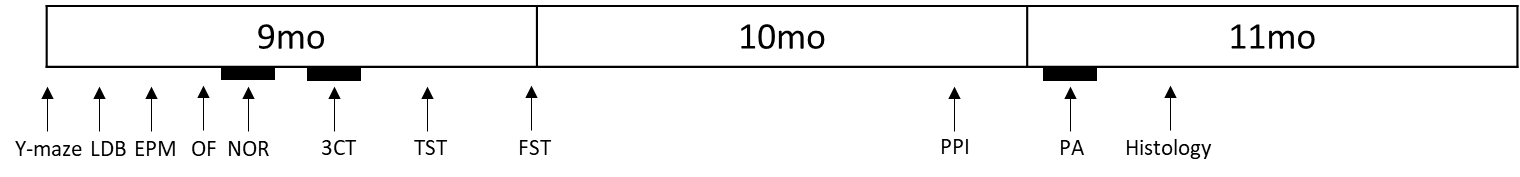


Figure S1. The experimental schedule.

Y-maze, LDB, EPM, OF, NOR, 3CT, TST, and FST were conducted with 9-months-old mice. PPI was conducted at the age of 10 months. PA and histological examination were performed at the age of 11 months.


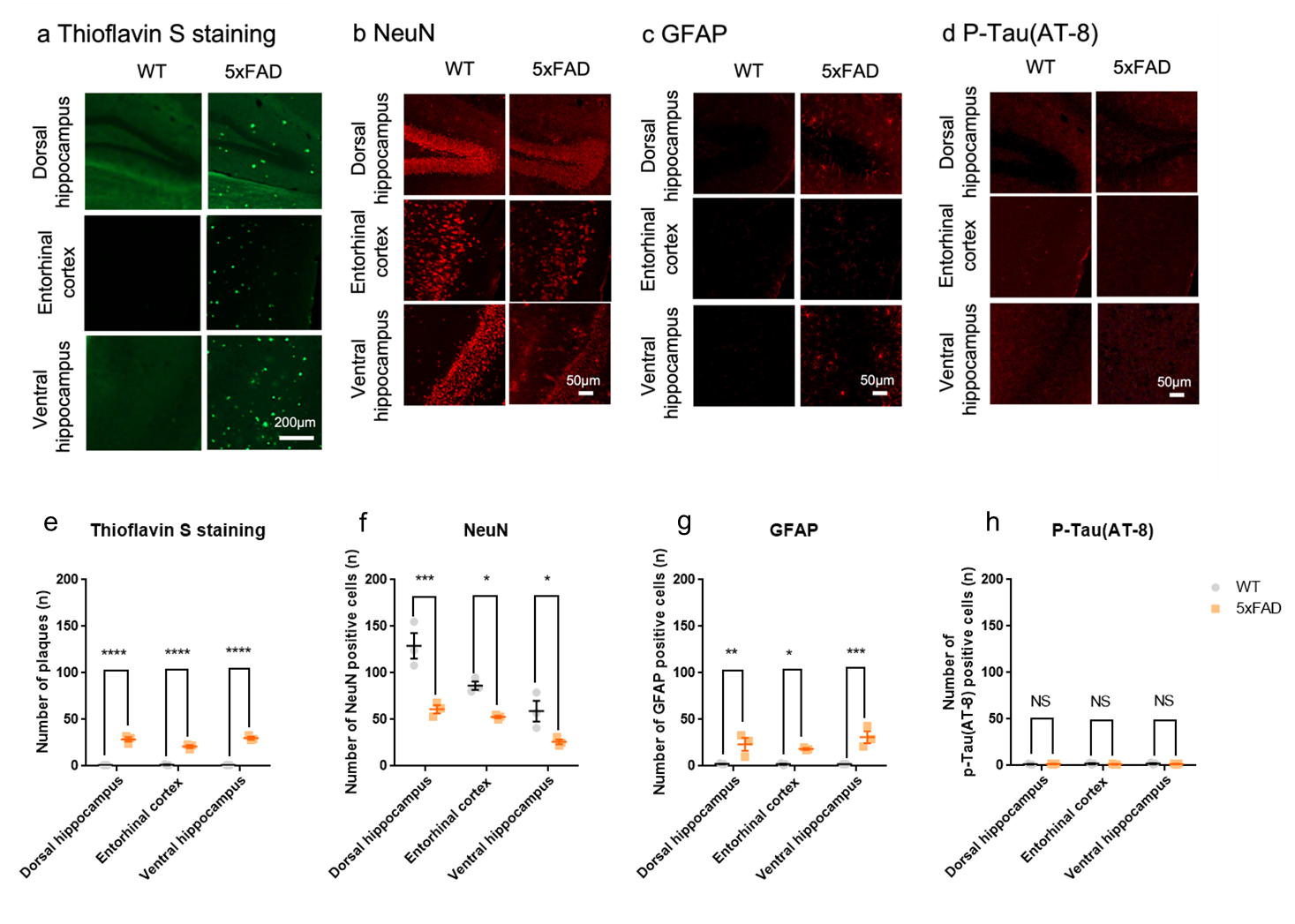


Figure S2. Alzheimer’s disease-related pathological phenotypes in the hippocampal formation of the 5xFAD mice at 11 months of age.

**a** Representative coronal sections of the dorsal hippocampus, entorhinal cortex, and ventral hippocampus from 11-month-old WT and 5xFAD mice stained with Thioflavin S. Scale bar, 200µm. **b-d** Representative coronal sections of dorsal hippocampus, entorhinal cortex, and ventral hippocampus from the WT and 5xFAD mice stained with antibodies for **b** NeuN, **c** GFAP, and **d** phosphorylated tau (P-Tau). **e-g** In the 5xFAD mice, the number of plaques and GFAP positive cells were significantly increased compared with WT mice, but the number of NeuN positive cells was significantly decreased with WT mice in the dorsal hippocampus, entorhinal cortex, and ventral hippocampus. **h** However, 5xFAD showed no significant difference of p-tau positive cell in the dorsal hippocampus, entorhinal cortex, and ventral hippocampus as compared with the WT mice. All data are given as means ± SEM (n=3 for WT, 3 for 6xTg; One-way ANOVA, *p<0.05,**p<0.01, ***p<0,001, ****p<0.0001). Scale bar, 200µm for **a**, and 50µm for **b-d**.


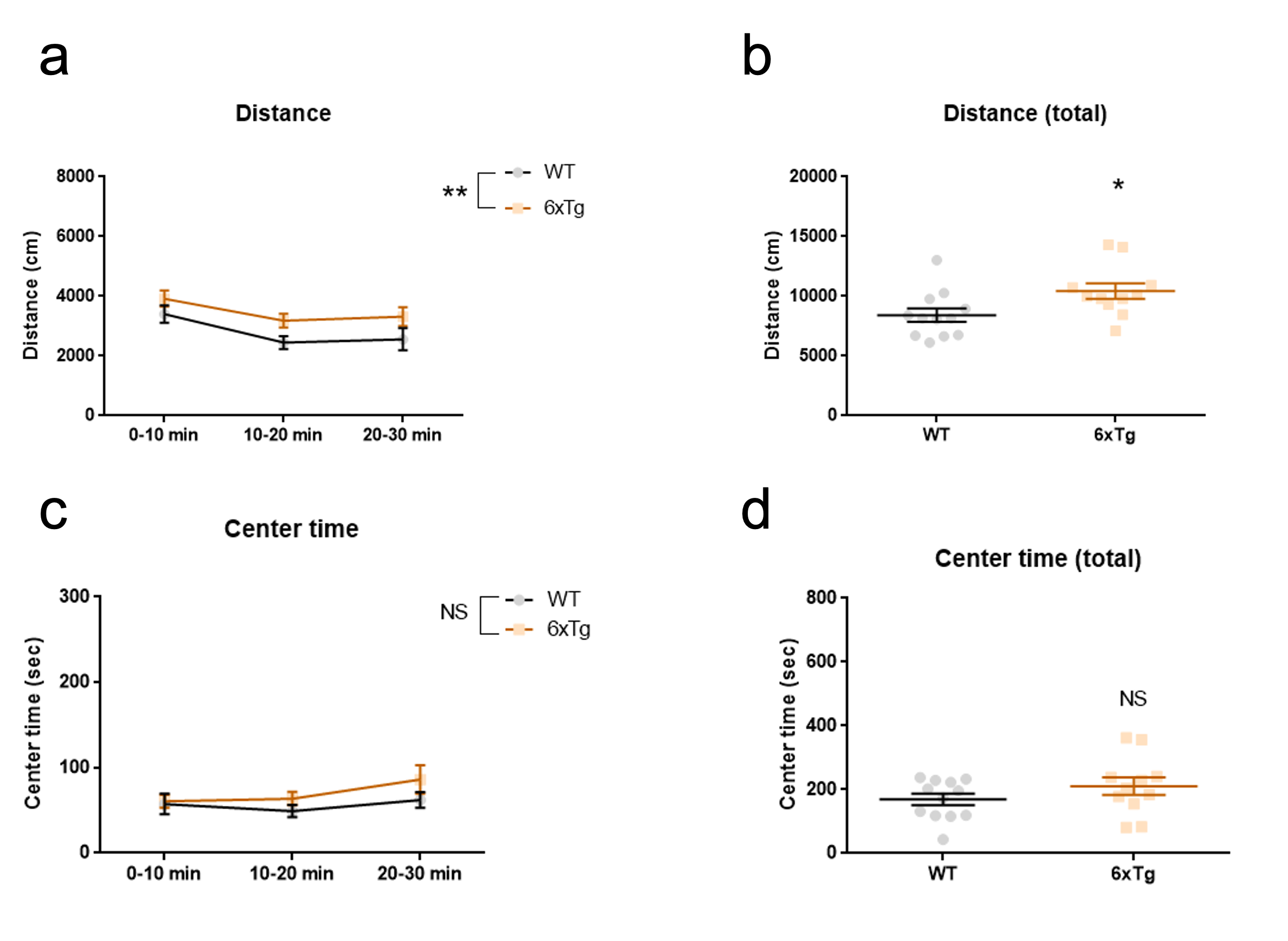


Figure S3. Increase in the general locomotor activity in 9-months-old 6xTg mice in the OF.

**a-b** The 6xTg covered more distance than did the WT, indicating increased locomotor activity. (n=12 for WT, 11 for 6xTg; Two-way ANOVA, *p<0.05, p<0.01). **c-d** The 6xTg and WT displayed similar time spent in the center zone of the open field (n=12 for WT, 11 for 6xTg; Two-way ANOVA, *p<0.05). All data are given as means ± SEM.


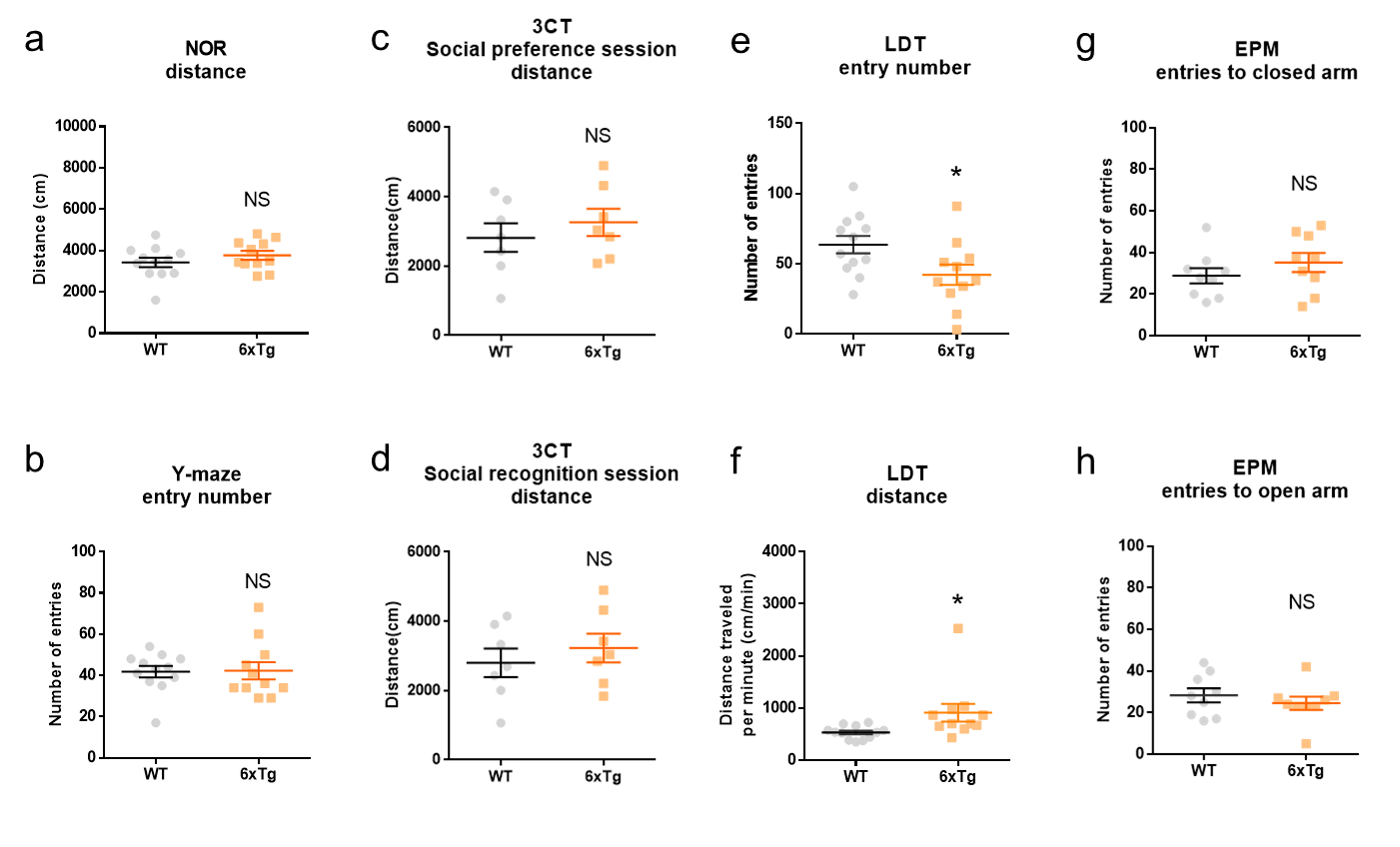


**Figure S4. Generally unchanged activity during the cognitive and anxiety tests in the 6xTg mice.**

**a** The 6xTg showed no significant difference in the distance traveled compared with the WT mice during NOR (n=12 for WT, 11 for 6xTg; Student’s t-test). **b** The 6xTg showed no significant difference in the number of entries made to each arm during Y-maze compared with the WT mice (n=12 for WT, 11 for 6xTg; Student’s t-test). **c-d** The 6xTg showed no significant difference in the distance covered in the 3CT compared with the WT mice (n=7 for WT, 7 for 6xTg; Student’s t-test). **e** The 6xTg showed reduction in the entries made to the light compartment in the LDT compared with the WT mice (n=12 for WT, 10 for 6xTg; Student’s t-test, *p<0.05). **f** The distance covered per minute in the light compartment during the LDT was significantly elevated in the 6xTg compared to the WT (n=12 for WT, 10 for 6xTg; Student’s t-test, *p<0.05). **g-h** The 6xTg showed no significant difference in the arm entries compared with WT mice in the EPM (n=9 for WT, 9 for 6xTg; Student’s t-test). NS, not significant. All data are given as means ± SEM.
